# Nrf2 Deficiency Unmasks the Significance of Nitric Oxide Synthase Activity for Cardioprotection

**DOI:** 10.1155/2018/8309698

**Published:** 2018-04-30

**Authors:** Ralf Erkens, Tatsiana Suvorava, Thomas R. Sutton, Bernadette O. Fernandez, Monika Mikus-Lelinska, Frederik Barbarino, Ulrich Flögel, Malte Kelm, Martin Feelisch, Miriam M. Cortese-Krott

**Affiliations:** ^1^Cardiovascular Research Laboratory, Division of Cardiology, Pulmonology and Vascular Medicine, Medical Faculty, Heinrich Heine University, Moorenstrasse 5, 40225 Düsseldorf, Germany; ^2^Clinical & Experimental Sciences, Faculty of Medicine, University of Southampton, Tremona Road, Southampton SO166YD, UK; ^3^NIHR Southampton Biomedical Research Centre, University Hospital Southampton NHS Foundation Trust, Tremona Road, Southampton SO166YD, UK; ^4^Department of Molecular Cardiology, Heinrich Heine University, Universitaetsstr. 1, 40225 Düsseldorf, Germany; ^5^Cardiovascular Research Institute Düsseldorf (CARID), Medical Faculty, Heinrich Heine University, Moorenstrasse 5, 40225 Düsseldorf, Germany

## Abstract

The transcription factor nuclear factor (erythroid-derived 2)-like 2 (Nrf2) is a key master switch that controls the expression of antioxidant and cytoprotective enzymes, including enzymes catalyzing glutathione de novo synthesis. In this study, we aimed to analyze whether Nrf2 deficiency influences antioxidative capacity, redox state, NO metabolites, and outcome of myocardial ischemia reperfusion (I/R) injury. In Nrf2 knockout (Nrf2 KO) mice, we found elevated eNOS expression and preserved NO metabolite concentrations in the aorta and heart as compared to wild types (WT). Unexpectedly, Nrf2 KO mice have a smaller infarct size following myocardial ischemia/reperfusion injury than WT mice and show fully preserved left ventricular systolic function. Inhibition of NO synthesis at onset of ischemia and during early reperfusion increased myocardial damage and systolic dysfunction in Nrf2 KO mice, but not in WT mice. Consistent with this, infarct size and diastolic function were unaffected in eNOS knockout (eNOS KO) mice after ischemia/reperfusion. Taken together, these data suggest that eNOS upregulation under conditions of decreased antioxidant capacity might play an important role in cardioprotection against I/R. Due to the redundancy in cytoprotective mechanisms, this fundamental antioxidant property of eNOS is not evident upon acute NOS inhibition in WT mice or in eNOS KO mice until Nrf2-related signaling is abrogated.

## 1. Introduction

Imbalanced redox equilibria are a hallmark of many pathological processes [1, 2]. An important mechanism by which cells adapt to an altered redox status as a result of a higher oxidative burden, or a compromised reductive capacity, or both, is transcriptional upregulation of a battery of cytoprotective genes. The same genes are also central to the supply with intracellular reducing equivalents required to maintain redox homeostasis and the detoxification of damaging electrophilic by-products of oxidants. The transcription factor Nrf2 is a key master regulator of the expression of genes, encoding antioxidant, detoxifying, and cytoprotective molecules such as heme oxygenase 1 (HO-1), SOD, glutathione S-transferase, glutamate cysteine ligase (an enzyme critical to glutathione biosynthesis), and NADPH quinone oxidoreductase 1 [3–6]. These genes contain the antioxidant response cis-element (ARE) in their promoters, which is a binding site for Nrf1 and Nrf2 transcription factors. Under steady-state conditions, Nrf2 remains sequestered in the cytoplasm by binding to Kelch-like ECH-associated protein 1 (Keap1). Oxidants and electrophiles induce the release of Nrf2 from the cytosolic complex by oxidation of cysteines within Keap1 and phosphorylation at specific sites [7], thus allowing Nrf2 to shuttle into the nucleus where it heterodimerizes with specific cofactors and coordinates upregulation of cytoprotective genes. Nrf1 serves complementary, but distinct functions including regulation of cell growth and metabolism, heme biosynthesis, and mitochondrial function [8].

Dysfunction in Nrf2-dependent gene regulation has been implicated in the pathogenesis of myocardial and renal ischemia, inflammatory disorders, cancer, and aging [9–12]. Ischemia/reperfusion (I/R) increased Nrf2 dissociation from Keap1, resulting in Nrf2 translocation into the nucleus, binding to the ARE, and activation of phase II detoxifying and antioxidant genes [9]. The Nrf2/ARE pathway affects cell survival through a variety of mediators, including apoptotic proteins such as Bcl-2 and Bax [13] and phase II enzymes such as HO-1 [14]. Moreover, Nrf2 is essential to successful ischemic preconditioning: two cycles of ischemic preconditioning did not result in cardiac protection in the absence of Nrf2 [2]. Likewise, several Nrf2 activators including glucocorticoids [15], endogenous prostaglandin D2 [16], and hydrogen sulfide (H_2_S) [17, 18] are cardioprotective in a Nrf2-dependent manner since this cardioprotection was lost in Nrf2 knockout (Nrf2 KO) mice.

Our recent study revealed that Nrf2 KO mice show cardiac hypertrophy, left ventricular diastolic dysfunction, and impaired Ca^2+^ homeostasis [19]. However, we found that vascular function in Nrf2 KO mice was fully preserved via a compensatory upregulation of eNOS. Considering the potent antioxidant and cardioprotective effects of nitric oxide (NO), we surmised that eNOS upregulation in Nrf2 KO mice may affect outcome after acute myocardial infarction. Specifically, we hypothesized that upregulation of eNOS may influence the degree of myocardial I/R injury under conditions of Nrf2 deficiency given the established significance of this transcription factor for cytoprotection and redox regulation.

To test this hypothesis, we first characterized overall thiol and NO metabolic status of Nrf2 KO mice. In a second step, we subjected Nrf2 KO mice to 30 min occlusion of the left anterior descending artery (LAD) followed by 24 h of reperfusion, analyzed infarct size and myocardial function in the presence and absence of a NOS inhibitor, and compared them with wild type (WT) and eNOS KO mice. We found that Nrf2 KO mice show decreased antioxidant capacity (GSH synthesis), yet display preserved redox status and levels of NO metabolites. Moreover, we found an eNOS-dependent cardioprotection against I/R injury in Nrf2 KO mice, which was abrogated by treatment with a NOS inhibitor. Cardioprotection by eNOS-derived NO was neither evident in WT mice treated with a NOS inhibitor nor in eNOS KO mice. Our data suggest that upregulation of eNOS and preserved NO bioavailability protects against myocardial I/R injury whenever antioxidant capacity is compromised like in Nrf2 KO mice. Intriguingly, due to the redundancy in cytoprotective mechanisms, this fundamental cardioprotective property of eNOS-derived NO is not evident upon acute NOS inhibition in WT or eNOS KO mice until Nrf2-related signaling is impaired.

## 2. Materials and Methods

### 2.1. Materials

Unless otherwise specified, chemicals were purchased from Sigma-Aldrich Chemie/Merck (Deisenhofen, Germany). Materials for Western blotting were purchased from Life Technologies (Invitrogen, Darmstadt, Germany).

### 2.2. Mice

Nrf2 KO/C57BL/6J (BRC number 01390) mice were obtained from Riken (Koyadai, Tsukuba, Ibaraki, Japan) and crossed for more than 10 generations with C57BL/6J. C57BL/6J mice were purchased from Janvier Labs (Le Genest-Saint-Isle, France) and used as WT mouse controls. eNOS KO mice on C57BL/6J background were generously provided by Dr. Axel Gödecke (Heinrich Heine University of Düsseldorf, Germany) [20]. Transgenic mice were bred and housed in the animal facility of the Heinrich Heine University, and male 5-6-month-old mice were used for experiments. All experiments were approved by the North Rhine-Westphalia State Agency for Nature, Environment and Consumer Protection and performed according to the guidelines for the use of experimental animals as given by German law.

### 2.3. Collection of Blood and Tissues from Nrf2 KO, eNOS KO, and WT Mice

For the analysis of thiols and NO metabolites, blood and organs were collected as described previously [21]. Briefly, mice were anesthetized with isoflurane (2.0%) and killed by exsanguination. Blood was transferred immediately into tubes containing N-ethylmaleimide (NEM)/EDTA (10 : 1 *v*/*v*), dissolved in phosphate-buffered solution (PBS) at pH 7.4 (final concentrations: 10 mM NEM, 2 mM EDTA), and centrifuged immediately for 3 min at 3000*g*. Organs were harvested after 1 min of perfusion with ice cold 10 mM NEM/2 mM EDTA in PBS pH 7.4, blotted dry on filter paper, weighed, snap frozen in liquid nitrogen, and kept at −80°C until later analysis. For Western blot analysis, organs were perfused with PBS pH 7.4 only.

### 2.4. Determination of Low-Molecular-Weight Thiols and Sulfide by Liquid Chromatography-Mass Spectrometry

Low-molecular-weight thiols and sulfide were measured as their NEM adducts using a Waters Acquity ultra-high pressure liquid chromatography (UHPLC) system coupled with a Xevo triple quadrupole (TQ-S) mass spectrometer. The UHPLC separation used an Acquity UPLC CSH C_18_ (1.7 *μ*m), 2.1 × 100 mm column. Gradient elution was used with the mobile phases consisting of H_2_O with 5 mM ammonium formate and 95% acetonitrile (ACN) (eluent A) and 5% H_2_O with 5 mM ammonium formate (eluent B). An injection volume of 5 *μ*l was used with a column temperature of 30°C and a flow rate of 0.2 ml/min. The gradient started at 95% A, decreasing down to 40% over 5 min, returning to 95% A by 6 min and allowing the column to equilibrate for another min, resulting in a total run time of 7 min. The elution times of each compound measured were (in min) 0.94 (GSSG), 1.80 (GluCys), 2.00 (GSH), 2.11 (Cys), 2.26 (CysGly), 2.35 (HCys), and 4.80 (sulfide).

### 2.5. Determination of NO Metabolites

Nitrosated (S-nitroso and N-nitroso) products (RXNO), NO heme, and nitrosyl species were quantified by gas phase chemiluminescence as described [21]. For nitrite/nitrate analysis, NEM-treated samples were deproteinized with ice-cold methanol (1 : 1 *v*/*v*), cleared by centrifugation and subjected to analysis by high pressure liquid chromatography using a dedicated nitrite/nitrate analyzer (ENO20, Eicom) as described [22].

### 2.6. Determination of eNOS Expression by Western Blot Analysis

Lysis of the heart and aorta, sample preparation, and Western blot analysis and detection were carried out as described [19]. Briefly, organs were lysed in RIPA buffer (1% NP40, 0.5% sodium deoxycholate, and 0.1% SDS in PBS pH 7.4) containing a cocktail of protease and phosphatase inhibitors (Pierce), homogenized at 4°C by using Tissue Ruptor (Qiagen, Hilden, Germany), sonicated for 3 min at 4°C, and centrifuged at 4000 ×g for 10 min at 4°C. Total protein concentration of the supernatant was determined by Lowry assay. Samples were loaded in 7% NuPAGE Tris-Acetate precast gels (Invitrogen) and transferred onto PVDF membrane Hybond P (Amersham Biosciences, Munich, Germany). The membranes were blocked for 2 hours with 5% BSA (Bio-Rad) in T-TBS (10 mM Tris, 100 mM NaCl, 0.1% Tween) and incubated overnight at 4°C with a mouse anti-eNOS (1 : 250, custom made from number 624086 anti-eNOS/NOS type III antibody, stock: 1 mg/ml in PBS pH 7.4, BD Bioscience, Erembodegem, Belgium) or monoclonal mouse anti-*α*-tubulin (1 : 5000, number T6199, Sigma-Aldrich) in T-TBS. After washing for 1 hour in T-TBS, the membranes were incubated with HRP-conjugated goat anti-mouse or anti-rabbit secondary antibodies (1 : 5000; BD Biosciences), and bands were detected using Amersham ECL Select Western Blotting Detection Reagent (number RPN2235, GE Healthcare) and Image Quant (GE Healthcare). Densitometry was carried out using Image Studio Lite software (LI-COR Biotechnology, Lincoln, NE, USA). Detection and quantification of the bands was compared within the linear range of the respective method of analysis.

### 2.7. Myocardial Ischemia and Reperfusion Protocol

Myocardial ischemia was induced by 30 min of ischemia followed by 24 h of reperfusion in WT, Nrf2 KO, and eNOS KO mice as previously described [23]. Briefly, after skin incision, median sternotomy, and pericardiotomy, the left anterior descending coronary artery was ligated halfway from base to apex with a 7-0 silk suture. Myocardial ischemia was induced for 30 min and confirmed by ST elevation on the electrocardiogram recorded during the ischemic phase and the first minute after reperfusion. Infarct size was evaluated by staining with 2,3,5-triphenyltetrazolium chloride (TTC). Briefly, the hearts were excised and perfused with 0.9% NaCl. The left anterior descending artery was reoccluded in the same location, and 1% Evans Blue dye was injected into the aortic root to delineate the area at risk (AAR) from not-at-risk area. The hearts were frozen at −20°C for 60 min and serially sectioned in 1 mm slices; each slice was weighed and incubated in 1% TTC for 5 min at 37°C. AAR and nonischemic areas were evaluated by computer-assisted planimetry. The size of the myocardial infarct is expressed as a percentage of the infarcted tissue area compared to the total AAR. A subgroup of mice received a continuous intraperitoneal (i.p.) infusion of the NOS inhibitor S-ethylisothiourea hydrobromide (ETU) (1.3 mmol/kg/min) during the ischemic period and in the early reperfusion. Efficient NOS inhibition using this protocol was independently verified by analyzing the concomitant increase in systolic and diastolic blood pressure 15 min after ETU administration as assessed invasively by Millar catheter in anesthetized WT and Nrf2 KO mice [19].

### 2.8. Analysis of Cardiac Function by High-Resolution Ultrasound and Cardiac Magnetic Resonance Tomography (cMRT)

Transthoracic echocardiography was performed as previously described [19]. Left ventricular volume (LV), end-systolic volume (ESV), and end-diastolic volume (EDV), LV ejection fraction (EF), fractional shortening (FS), cardiac output (CO), and stroke volume (SV) were calculated. LV diastolic function was assessed by analysis of the characteristic flow profile of the mitral valve Doppler which was visualized in apical four-chamber view, as described [19]. Cardiac magnetic resonance tomography imaging data were recorded by a Bruker AVANCEIII 9.4 T wide bore NMR spectrometer (Bruker, Rheinstetten, Germany) at 400.13 MHz operated by ParaVision 5.1. Images were acquired using the Bruker microimaging unit Micro 2.5 with actively shielded gradient sets (1.5 T/m) and a 25 mm birdcage resonator, as previously described [24].

### 2.9. Statistical Analysis

Statistical calculations for each experiment and the number of animals are stated in Results and figure legends. Statistical comparisons between groups were calculated by either Student's *t*-tests (for 2 groups) or Tukey's multiple comparison post hoc test following one-way ANOVA (for more than two groups). Browne-Forsythe test was performed to check for homoscedasticity of our samples. Post hoc tests were run only if F was not different between the groups and there was no significant variance inhomogeneity. *p* < 0.05 was considered statistically significant.

## 3. Results

### 3.1. Decreased Antioxidant Reserve Capacity with Preserved Redox State in Nrf2 KO Mice

**Scheme 1:**
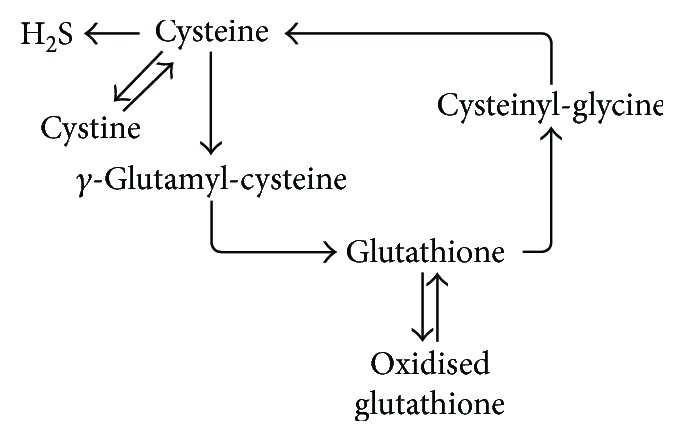
Simplified schematic representation of the main metabolic pathways involved in the production and degradation of glutathione in mammalian tissues.

It is well known that mice lacking the transcription factor Nrf2 show decreased expression of enzymes involved in glutathione biosynthesis, especially in the liver [25, 26]. To address the question how a lack of Nrf2 affects circulating and tissue thiol metabolic status in the heart and other organs, we assessed the concentration of reduced and oxidized glutathione and some of its precursors in blood and tissues of Nrf2 KO and WT mice. A schematic representation of the main metabolic pathways regulating GSH synthesis is provided in Scheme 1. With the exception of red blood cells, we found that a lack of Nrf2 significantly affects total GSH levels in plasma and all analyzed organs (Table 1), indicating a decreased antioxidant reserve capacity in Nrf2 KO mice as compared to WT mice. Interestingly, the GSH/GSSG ratio in the heart and aorta was similar in Nrf2 KO and WT mice, demonstrating that the redox state in these compartments of Nrf2-deficient mice is fully preserved. We also found that steady-state concentrations of the glutathione precursors, homocysteine, cysteine, and *γ*-glutamylcysteine, and that of the decomposition product cysteinylglycine were significantly decreased in all organs and in plasma but not in RBCs of Nrf2 KO mice (Table 1). By contrast, RBCs appear to have a peculiar resistance to changes in total thiol levels and displayed a significant increase in their GSH/GSSG ratio under conditions of Nrf2 deficiency. The present study offers evidence of an altered antioxidant capacity in Nrf2 KO mice compared to WT mice, but further examination with a larger cohort of mice is necessary to confirm this finding.

**Figure 1:**
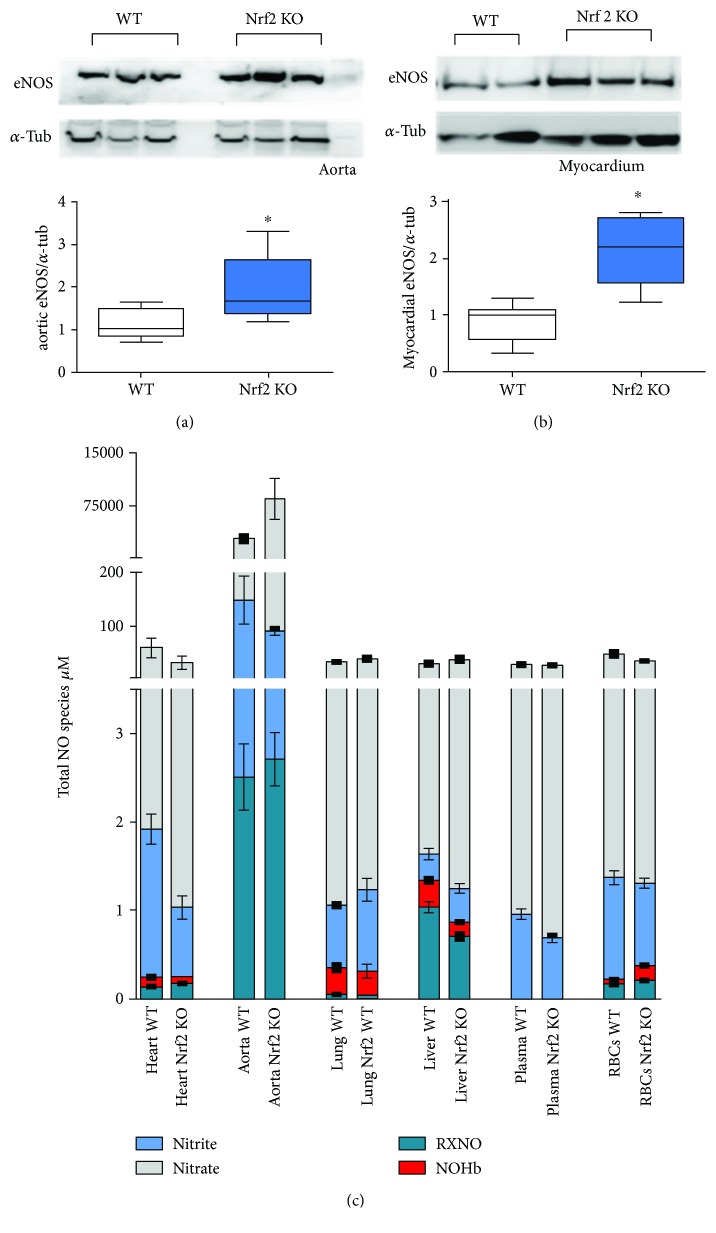
Upregulation of eNOS and preserved total levels of NO metabolites in Nrf2 KO mice. (a) Representative Western blot of eNOS (upper panel) standardized to alpha-tubulin (lower panel) and densitometric analysis of eNOS expression in aorta and (b) heart of WT and Nrf2 KO mice detected by Western blot (*n* = 5 per group, means ± quartiles, ^∗^ *p* < 0.05, *t*-test). (c) Total NO species in different tissues of Nrf2 KO and WT mice (*n* = 5 per group, means ± SEM), (for statistical comparisons and SD please refer also to Table 2).

Next, we aimed to analyze how the changes in the thiol metabolic status of Nrf2 KO mice may affect NO metabolism. In good agreement with data published elsewhere [19], we observed an upregulation of eNOS protein in myocardial and vascular tissue of Nrf2 KO mice (Figures 1(a) and 1(b)). Importantly, previous analysis of these mice revealed that the upregulated eNOS in Nrf2 KO mice was functionally active as evidenced by increased flow-mediated vasodilation in vivo and increased carbachol-induced cGMP reposes in aortic rings from Nrf2 KO mice as compared to WT mice [19]. Here, we found that the total levels of nitrite, nitrate, and nitroso species in all compartments were not significantly different between WT and Nrf2 KO mice (Figure 1(c)), except in the aorta. Of note, nitrosyl-hemoglobin levels in RBCs were increased in Nrf2 KO mice as compared to WT mice (Table 2), consistent with the notion of an enhanced NO availability secondary to eNOS upregulation in the vasculature. While total levels of NO-derived species remain essentially unchanged between groups, there are distinct distributions for individual species across groups. Determining whether these trends bear statistical significance, particularly in the case for nitrosation products (RXNO) would require a follow-up study with a larger cohort of mice.

Taken together, these data suggests that Nrf2 KO mice have greatly impaired activity for glutathione synthesis and therefore a diminished antioxidant reserve capacity in all compartments except the erythrocytes, yet overall redox status and total NO metabolite concentrations are well preserved.

### 3.2. Nrf2 KO Mouse Hearts Are More Resilient toward Myocardial I/R Injury, and This Extra Protection Is Dependent on NOS Activity

**Figure 2:**
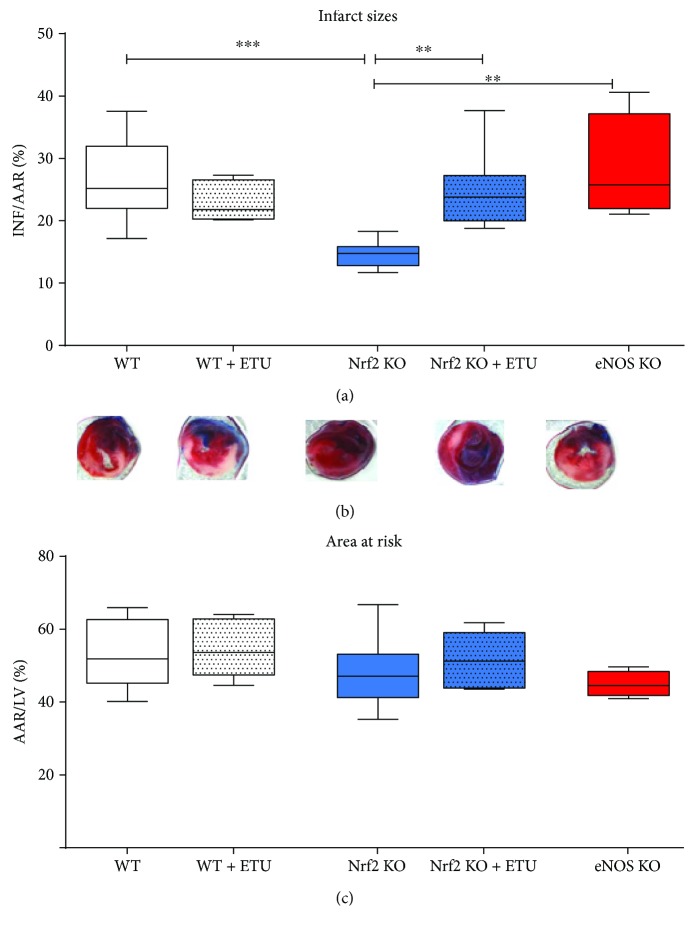
NOS-dependent decrease of infarct size in Nrf2 KO mice. (a) Infarct sizes (INF) per area at risk (AAR). Nrf2 KO mice showed a significant decrease in infarct sizes as compared to WT mice. Application of the NOS inhibitor ethylthiourea (ETU) in WT mice did not affect infarct size, whereas ETU application in Nrf2 KO mice increased I/R injury demonstrating the cardioprotective role of NOS activity despite a compromised antioxidative reserve capacity in Nrf2 KO mice. eNOS KO mice showed no differences in I/R injury as compared to WT and WT + ETU mice. WT mice: *n* = 9, Nrf2 KO mice: *n* = 8, eNOS KO mice: *n* = 4‐5; Browne-Forsythe test *p* = 0.27, means ± quartiles, one-way ANOVA ^∗∗^ *p* < 0.01, ^∗∗∗^ *p* < 0.001. (b) Representative TTC stained heart sections of each strain 24 h after AMI. (c) Comparable values of area at risk/left ventricle demonstrate the reproducibility of the I/R surgery (Browne-Forsythe test *p* = 0.24, means ± quartiles, one-way ANOVA, ns).

To study the effects of a diminished antioxidant reserve capacity on the susceptibility to myocardial ischemia/reperfusion injury, Nrf2 KO mice were subjected to 30 min occlusion of the left anterior descendent (LAD) artery followed by 24 h of reperfusion, and infarct size and area at risk (AAR) were compared between WT and eNOS KO mice (Figure 2). Unexpectedly, infarct size per AAR was not increased but significantly decreased in Nrf2 KO mice as compared to WT mice (14.47 ± 0.7% versus WT mice: 26.68 ± 2.1% (Figures 2(a) and 2(b)), *n* = 9 each, *p* < 0.001), while the AAR of the left ventricles did not differ between these strains (Figure 2(c)).

We reasoned that the enhanced cardioprotection against I/R injury in Nrf2 KO mice may be a result of compensatory eNOS upregulation. To test this assumption, we treated mice with the NOS inhibitor ETU via continuous i.p. infusion during ischemia and throughout the first 5 minutes of reperfusion. We found that NOS inhibition did not affect infarct size in WT mice, while it significantly increased infarct size in Nrf2 KO mice when compared to untreated controls (Figures 2(a) and 2(b)), such that there was no significant difference between infarct size in the ETU-treated WT and Nrf2 KO mouse hearts. As a further control, the effects of I/R injury were also tested in mice globally deficient for eNOS. Similar to what we observed after acute NOS inhibition in the WT mice, we found no difference in infarct size between eNOS KO and WT mice (Figure 2(a), red boxes).

Taken together, these data demonstrate that upregulation of eNOS in the Nrf2 KO mouse heart accounts for the improved protection of Nrf2 KO mouse myocardium from I/R injury-induced cell death; this cardioprotective property of eNOS is not evident upon acute NOS inhibition in WT or chronic eNOS deficiency in eNOS KO mice but is unmasked when Nrf2-related signaling is impaired.

### 3.3. In Nrf2 KO Mouse, Systolic and Diastolic Myocardial Functions after Acute Myocardial Infarction (AMI) Are Preserved by NOS

**Figure 3:**
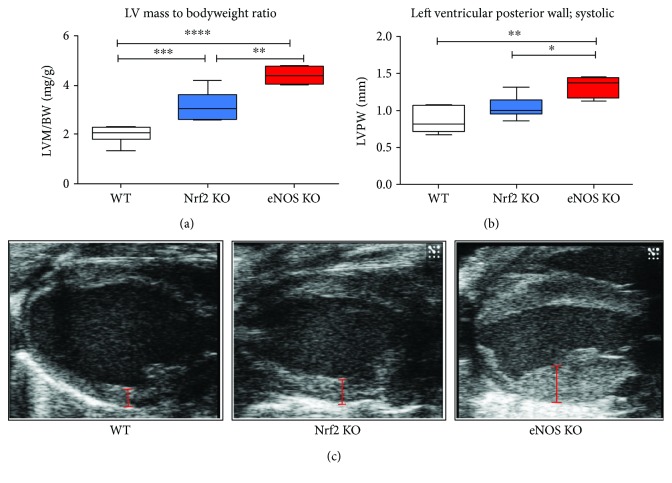
Cardiac hypertrophy in Nrf2 KO and eNOS KO mice. (a) LV mass normalized to body weight illustrates cardiac hypertrophy in both Nrf2 KO and eNOS KO mice as compared to WT mice. (b) Diameter of left ventricular posterior wall measured at the end of systole highlights the increase in myocardial hypertrophy in both KO mice. (c) Representative B-mode pictures of the heart from different mouse strains visualize the extent of cardiac hypertrophy. Red bars show the diameter of the left ventricular posterior wall. WT mice: *n* = 8, Nrf2 KO mice: *n* = 9, eNOS KO mice: *n* = 4; Browne-Forsythe test *p* > 0.05, means ± quartiles, one-way ANOVA ^∗^ *p* < 0.05, ^∗∗^ *p* < 0.01, ^∗∗∗^ *p* < 0.001, ^∗∗∗∗^ *p* < 0.0001.

**Figure 4:**
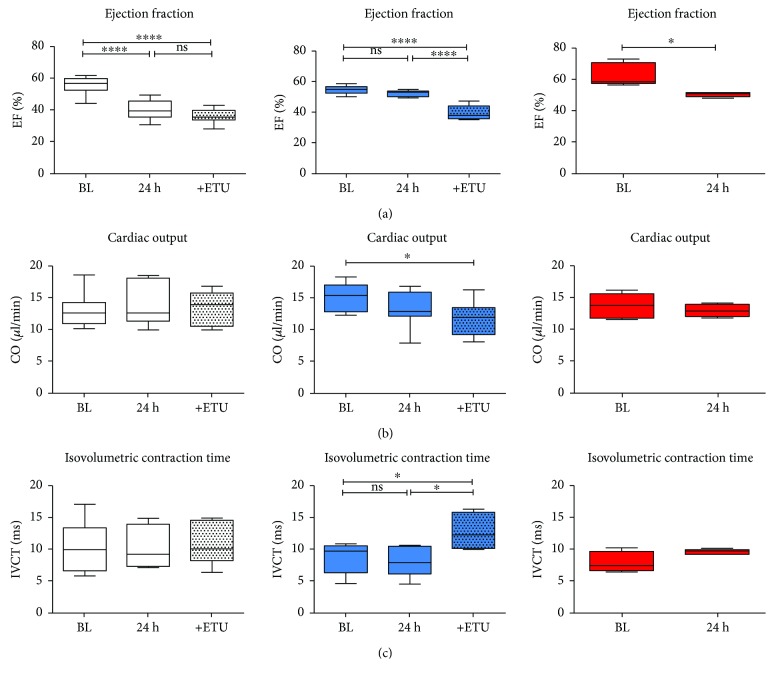
Left ventricular systolic function following AMI in Nrf2 KO mice is preserved by NOS. (a) In WT (white box plots) and eNOS KO (red box plots) mice, ejection fraction (EF) was significantly decreased 24 h after AMI, whereas it was essentially unchanged in Nrf2 KO mice (blue box plots). ETU application resulted in an impairment of systolic function in Nrf2 KO mice, but not in WT mice, indicating NOS-dependent preservation of cardiac function in Nrf2 KO mice as compared to WT mice. (b) Cardiac output (CO) was significantly decreased after ETU application in Nrf2 KO mice indicating eNOS-dependent preservation of systolic function after I/R. (c) IVCT was increased after ETU application in Nrf2 KO mice, but not in WT mice. WT mice: *n* = 9, Nrf2 KO mice: *n* = 8; Browne-Forsythe test *p* > 0.05, means ± quartiles, one-way ANOVA, except for eNOS KO mice (red boxes): 4-5, unpaired *t*-test. ^∗^ *p* < 0.05 and ^∗∗∗∗^ *p* < 0.0001.

At baseline conditions, Nrf2 KO mice display well-preserved left ventricular systolic function as measured by cMRT (Table 3), while their left ventricular diastolic function is significantly impaired (Supplementary Table 1). These results fully confirm our previous echocardiographic analyses in Nrf2 KO mice [19]. Of note, Nrf2 KO mice showed some degree of LV hypertrophy as evidenced by increase of the LV mass to body weight ratio (Figure 3(a)). Furthermore, ESV and EDV in Nrf2 KO mice were significantly higher than in WT mice (Supplementary Table 1). Mice lacking eNOS also suffer from cardiac hypertrophy (Figure 3), which was much more prominent than that of Nrf2 KO mice, but was not accompanied by any changes in systolic functional parameters (except for SV) as compared to WT mice (Figure 4, Supplementary Table 1).

Twenty-four hours after AMI, left ventricular systolic function was impaired in WT mice as evidenced by significantly reduced ejection fraction (EF) (Figure 4(a)). In contrast, in Nrf2 KO mice, EF was fully preserved after AMI as compared to baseline (Figure 4(a)). Interestingly, acute NOS inhibition with ETU in Nrf2 KO mice impaired EF after AMI, but did not affect EF in WT mice as compared to untreated mice (Figure 4(a); ETU treatment). Likewise, NOS inhibition significantly decreased CO in Nrf2 KO mice only, but did not affect it in WT mice, pointing to a role of NOS activity in preserving LV systolic function after AMI in Nrf2 KO mice but not in WT mice (Figure 4(b)). Consistent with the results obtained after NOS inhibition in WT mice, CO was not changed after myocardial I/R injury in eNOS KO mice as compared to baseline (Figure 4(b), red boxes). Furthermore, NOS inhibition in Nrf2 KO mice resulted in a significant impairment of cardiac contraction, as indicated by an increase in isovolumetric contraction time (IVCT) measured in apical four-chamber view using pulse wave Doppler (Figure 4(c), Supplementary Table 1, Nrf2 KO + ETU post I/R versus Nrf2 KO post I/R) while NOS inhibition in WT mice had no effect on IVCT, and there was no difference in IVCT in eNOS KO post I/R versus baseline values (Figure 4(c), red boxes).

**Figure 5:**
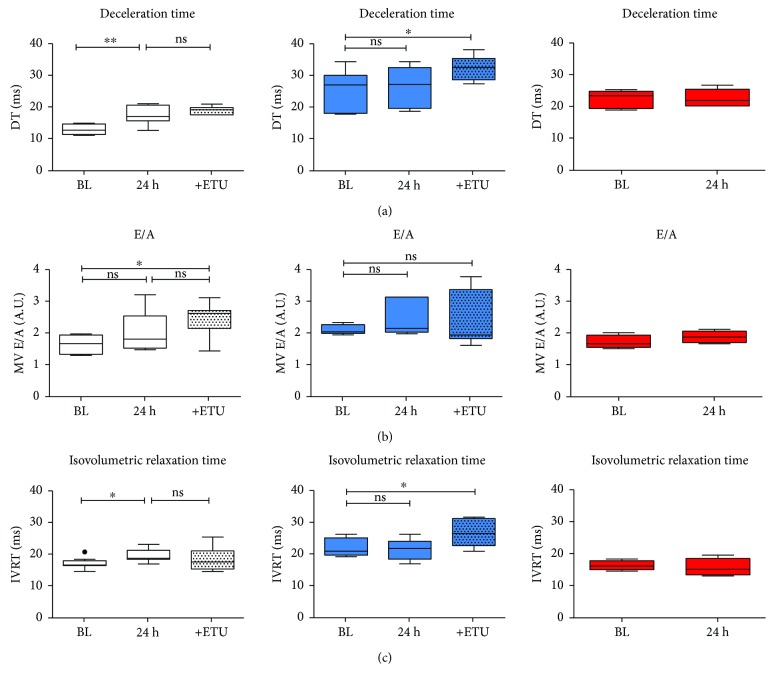
Nrf2 KO mice are protected against impairment of myocardial diastolic function following AMI. (a) I/R injury resulted in an impairment of diastolic function (evaluated with prolonged deceleration time (DT)), (b) E/A relation, and (c) relaxation time (IVRT) in WT mice (white box plots), whereas preexisting diastolic dysfunction was not aggravated in Nrf2 KO mice (blue box plots). In eNOS KO mice (A, red box plots), diastolic dysfunction evidenced by increased DT as compared to WT mice (Supplementary Table 1) was not further exacerbated after AMI. WT mice: *n* = 9, Nrf2 KO mice: *n* = 8; Browne-Forsythe test *p* > 0.05, means ± quartiles, one-way ANOVA ^∗^ *p* < 0.05, ^∗∗^ *p* < 0.01 except for eNOS KO mice *n* = 4‐5, unpaired *t*-test.

Assessment of LV diastolic function by mitral flow Doppler imaging showed that Nrf2 KO mice have significant diastolic dysfunction at baseline as evidenced by prolonged DT and IVRT and increased E/A ratio (Supplementary Table 1). Surprisingly, despite the prominent diastolic dysfunction and reduced capacity of myocardial relaxation, LV diastolic dysfunction was not further aggravated by AMI in Nrf2 KO mice as there were no changes in diastolic functional parameters (Figure 5), while E wave DT, IVRT, and total diastolic time were significantly impaired after AMI in WT mice (versus WT baseline, white boxes, Figure 5; Supplementary Table 1). Following administration of the NOS inhibitor, LV DT in Nrf2 KO mice was further impaired (Figure 5(a), red boxes), whereas NOS inhibition in WT mice did not affect DT (Figure 5(a), Supplementary Table 1). Similar to the effects of acute NOS inhibition, LV diastolic functional parameters were not changed post AMI in eNOS KO mice (Figure 5, red boxes); however, these mice exhibited LV diastolic dysfunction at baseline which was evidenced by almost twofold increased DT (Supplementary Table 1).

Taken together, these data suggest that Nrf2 KO mice, despite their decreased antioxidant reserve capacity, are more protected against AMI than WT mice, as evidenced by reduced infarct size, maintained systolic function and a lack of postischemic facilitation of LV diastolic dysfunction. Moreover, the results of NOS inhibition experiments revealed the increased infarct size, post AMI impairment of left ventricular systolic dysfunction, and increase of diastolic dysfunction in Nrf2 KO mice demonstrating that NOS-dependent signaling pathways play a key role in the protection of the heart against I/R injury under conditions of Nrf2 deficiency.

## 4. Discussion

The main finding of our study is that upregulation of eNOS mediates cardioprotection against I/R under conditions of decreased antioxidant capacity in mice lacking the transcription factor Nrf2. Specifically, we demonstrated that (1) Nrf2 KO mice have a severe decreased antioxidant reserve capacity, characterized by significantly decreased concentration of total glutathione, and of its precursors, but show fully preserved redox status and global NO bioavailability at baseline; (2) following myocardial I/R injury Nrf2 KO mice had a reduced infarct size and (in contrast to WT mice) showed a fully preserved systolic LV function; (3) inhibition of NO synthase activity at the onset of ischemia and during early reperfusion increased myocardial damage and induced systolic dysfunction in Nrf2 KO mice, but not in WT mice; (4) consistent with the results upon acute NOS inhibition in WT mice, infarct size in eNOS KO mice was not different from that in WT mice. Taken together, these data suggest that upregulation of eNOS under conditions of decreased antioxidant capacity due to Nrf2 deficiency likely plays an important role in cardioprotection against I/R injury, while this mechanism of cardioprotection is not evident after acute NOS inhibition in WT or in eNOS KO mice.

### 4.1. Decreased Antioxidant Capacity and Preserved NO Bioavailability in Nrf2 KO Mice

Nrf2 is known to play a central role in cellular stress response, adaptation, and resilience [1]. Accordingly, Nrf2 deletion has been shown to result in downregulation of phase 2 detoxifying enzymes (like glutathione S-transferase), antioxidant enzymes (like SOD), and enzymes responsible for glutathione de novo biosynthesis/thiol metabolism (like glutamate cysteine ligase and glutathione synthetase) as assessed in the liver and other organs including the heart [17, 27, 28]; however, a proportion of certain organ-specific Nrf2 target genes is also regulated by other transcription factors, like the aryl hydrocarbon receptor (AhR) [29]. We here provide evidence that thiol metabolism and total antioxidant capacity are strongly impaired in Nrf2 KO mice in all analyzed organs, and this translates into decreased concentrations of glutathione and its precursors, cysteine, homocysteine, and *γ*-glutamylcysteine. We also found that the changes in glutathione-related aminothiols are tissue-specific. Although this is consistent with the notion of differential expression of Nrf2 target genes among different organs, a larger cohort of animals is necessary to confirm our findings.

Interestingly, tissue redox status (i.e., the ratio of reduced/oxidized thiols in different organs) in Nrf2 KO mice is not significantly different from that of WT mice, as a result of compensatory alterations of multiple reduced and oxidized thiols. Consistent with a fully preserved redox status, the total levels of NO metabolites remain essentially unchanged between the groups. However, upon observation on these data, there are distinct distributions for individual species across groups. This is particularly the case for nitrosation products (RXNO), exhibiting a *p* value of 0.0603. Determining whether these trends bear statistical significance would require a follow-up study with a larger cohort of animals. Consistently to preserved levels of NO metabolites, we found that the expression of eNOS was upregulated both in the conduit arteries and in the heart of Nrf2 KO mice as shown by us previously [19] and in this study (which corresponds to increased eNOS-derived NO production with consecutive cGMP-mediated vascular responses). Moreover, this eNOS upregulation translates into elevated nitrosyl-hemoglobin concentrations in RBCs, a good indicator of increased NO bioavailability [21, 30].

Taken together, while the lack of Nrf2 induces a profound decrease in overall antioxidant reserve capacity in all organs, especially in the heart, redox status, total levels of NO metabolites, and NO bioavailability are fully preserved. Further studies with a larger cohort of mice and mice lacking both Nrf2 and eNOS will reveal the causal relationship between lack of Nrf2 and eNOS-derived NO formation to preserve antioxidant capacity and NO bioavailability.

### 4.2. Nrf2 KO Mice Are Protected against I/R in a NOS-Dependent Fashion

It is well known that the reoxygenation of the heart during reperfusion is associated with an increase in the production of superoxide and other reactive oxygen species by a variety of enzymes including xanthine oxidase, NADPH oxidases, and respiratory complexes of the mitochondrial electron transport chain. Under these conditions, Nrf2-dependent antioxidant enzymes and the pool of reduced thiols are thought to counterbalance the regional increase in oxidant formation, thereby protecting the myocardium from fatal damage. Thus, lack of Nrf2-dependent protection coupled with downregulation of antioxidant genes and reduced redox capacity of the myocardial and vascular tissues was expected to have detrimental effects in myocardial I/R injury. Moreover, as we previously demonstrated that Nrf2 KO mice have LV diastolic dysfunction (characterized by a decreased cardiac relaxation and impaired Ca^2+^ homeostasis; [19]), one might expect a further impairment of cardiac function in Nrf2 KO mice following AMI secondary to a reduced capacity of myocardial relaxation.

Surprisingly, we found that the Nrf2 KO mouse hearts were fully protected from I/R injury. Specifically, Nrf2 KO mice showed a significantly decreased infarct size and preserved LV systolic function after AMI as compared to WT mice. Furthermore, following I/R injury, we failed to detect any further decline of LV diastolic function in Nrf2 KO mice, which was evident in WT post AMI. The reduced infarct size after I/R injury in Nrf2 KO mice when compared to WT mice seems to be in contrast to previously published findings on an increased sensitivity of the Nrf2 KO mouse hearts to ischemic reperfusion injury [17]. A possible explanation for these contradictory findings might be the different strains of Nrf2 KO mice used in this and our study, specifically with regard to genetic background. In our study, we used Nrf2 KO mice on a fully backcrossed C57BL/6 background, while others studied Nrf2 KO mice on a SVB background [2, 17, 31]. Of note, Nrf2 KO mice on SVB background do not show LV diastolic dysfunction at baseline (but develop it only after transverse aortic constriction [32]), in addition, they displayed a lupus-like phenotype [33], but the expression of eNOS or NO metabolites were not investigated in these mice. Additional experiments, for example, future studies in Nrf2 KO mice crossbreed with the eNOS-deficient mice, might represent a better approach to study a role of eNOS in cardiovascular protection under conditions of chronic Nrf2 deficiency.

Importantly, the Nrf2 KO mice used in the present study are characterized by upregulation of a fully functional eNOS, which results in hypotension, preserved vascular function *in vivo* (FMD) and ex vivo (aortic ring relaxation), and increased cGMP production in response to pharmacological eNOS stimulation in Nrf2 KO mouse vessels while basal levels of cGMP were not changed [19]. Except of the classical NO-sGC-cGMP signaling, NO can also exert its regulatory effects on protein structure and function through S-nitrosation of cysteine thiols [34, 35]. Published data demonstrate eNOS-dependent S-nitrosation of many myocardial proteins including beta-arrestin, cardiac ion channels, and GPCR kinase-2, which influence heart contractility and myocyte survival and function [36–40]. S-Nitrosation of these proteins during myocardial I/R can attenuate apoptosis [41] and inflammation [39]. For instance, caspase-3 activity is known to be inhibited by protein S-nitrosation [42]. Furthermore, mitochondrial ROS production can be decreased during I/R, since S-nitrosothiols are able to modify complex I of the mitochondrial electron transfer change [43] and prevent opening of the mitochondrial permeability transition pore [44] and thus may represent cardioprotective action of upregulated eNOS in Nrf2 KO mice. In this study, we could not detect a significant change in total levels of nitroso species in the heart (although a clear trend to increased RXNO levels was observed) which might be due to the fact that the total detected RXNO levels include not only RSNO but also other nitroso species (e.g., RNNO).

Here, we demonstrate that the increase in eNOS activity plays an important fole in cardioprotection against AMI in Nrf2 KO mice. This is supported by our finding that in Nrf2 KO mice, acute NOS inhibition greatly increased infarct size and decreased systolic and diastolic function after I/R, while neither NOS inhibition in WT mice nor chronic global eNOS deficiency in eNOS KO mice affected these parameters. Therefore, eNOS upregulation in Nrf2 KO mice likely represents an important protective mechanism that not only protects against myocardial I/R injury but also against impairment of systolic function and the aggravation of diastolic dysfunction after AMI in these mice.

### 4.3. Role of eNOS-Derived NO in Cardioprotection

Although we found eNOS-derived NO-mediated cardioprotection in Nrf2 KO mice, neither acute NOS inhibition nor global loss of eNOS revealed any cardioprotective effect of NO in the WT mouse hearts. Specifically, eNOS KO mice [20] exhibit no differences in infarct size as compared to the WT mice, which is consistent with the results obtained by acute treatment of WT mice with the NOS inhibitor ETU. Previous reports on the role of eNOS in I/R showed controversial effects, depending on the eNOS KO mouse strain with decreased infarct size in the strain created by Shesely et al. [45] attributed to iNOS upregulation and increased infarct size and myocardial necrosis [46] in the eNOS KO mouse strain created by Huang et al. [47].

Similar to these two strains, the eNOS KO mouse strain used in the present study (created by Gödecke et al. [20]) shows sustained hypertension, decreased heart rate, increased SV, and cardiac hypertrophy, manifested by increased ventricular wall thickness and mass. However, LV systolic function is fully preserved under basal conditions, as parameters including EF, CO, and FS did not differ between WT and eNOS KO mice. Similar results were reported for the eNOS KO mouse strain generated by Shesely [48]. Likewise, myocardial infarct size and cardiac performance after AMI were not more severe in eNOS KO mice created by Gödecke et al. [20] as compared to WT mice. Accordingly, acute NOS inhibition by ETU in WT mice did neither affect LV function after AMI nor infarct size. Therefore, these data clearly demonstrate that lack of eNOS activity is fully compensated by hemodynamic changes and/or cardiac remodeling (on the long term), and the cardioprotective role of eNOS becomes evident only under conditions of a compromised redox reserve like in Nrf2 KO mice. However, the mechanisms of compensation in Nrf2 KO mice treated with NOS inhibitor might differ from the compensation mechanisms of mice chronically deficient for eNOS.

Our experimental setup did not allow us to distinguish between endothelial and other sources of eNOS upregulation in Nrf2 KO mice. However, it has been suggested that activity of endothelial NOS during I/R is of minor importance as compared to the cardiomyocyte-derived NO. Mice with cardiomyocyte-specific overexpression of eNOS displayed significantly decreased infarct size and better preserved cardiac function than mice with systemic eNOS overexpression [49]. In contrast, a study from our laboratory proposed a potential key role of the red blood cell eNOS in cardioprotection against I/R [50]. Specifically, we demonstrated that depletion of circulating blood eNOS increases the severity of myocardial infarction and LV dysfunction suggesting a modulating role of circulating eNOS in an acute model of myocardial I/R. Future studies using tissue-specific transgenic mice with knockin and knockout of key proteins of the eNOS-dependent pathway will help delineating a specific role for eNOS expressed in RBCs and eNOS expressed in other cell types, for example, in endothelium and in cardiomyocytes in conferring cardioprotection against I/R.

Taken together, cardioprotective effects in Nrf2 KO mice were abolished by inhibition of NOS activity in ischemia and early reperfusion, suggesting a role for eNOS in cardioprotection against I/R in Nrf2 deficiency. These data demonstrate that impaired antioxidant defense and decreased redox reserve in Nrf2 KO mice or decreased NO bioavailability (like in eNOS KO or after NOS inhibition) on their own do not affect myocardial damage after AMI; however, their combination significantly increases myocardial damage and is in line with several recent publications on protective effects of eNOS under conditions of oxidative stress [51, 52].

## 5. Conclusion

Here, we present experimental evidence that eNOS upregulation under conditions of a compromised antioxidant capacity plays a role in cardioprotection against I/R injury, while eNOS-mediated cardioprotection is not evident after acute NOS inhibition or in eNOS KO mice per se. Although there is overwhelming evidence for associations between oxidative stress and I/R injury, clinical trials consistently failed to demonstrate any cardioprotective effect of antioxidants [53–55]; moreover, high doses of antioxidants may be even harmful [56, 57]. On the contrary, interventions associated with increased eNOS protein expression and improvement of endothelial NO production such as regular exercise training [58], pharmacotherapy with statins [59], and treatment with inhibitors of the renin-angiotensin system [60] have provided superior cardiovascular prevention compared to approaches targeting oxidative stress by applying antioxidants [53–55, 61]. Our results on the beneficial role of eNOS upregulation in cardioprotection under conditions of Nrf2 deficiency and compromised antioxidant status support these data. These results might be also of special importance for the aged organisms, since Nrf2 signaling pathway may substantially contributes to the determination of health span and extension of longevity [62]; and diminished activity of Nrf2 resulting in reduced antioxidative potential, apoptosis, and/or necrosis of the aging myocardium has been reported [63, 64]. Whether the relationship between redox reserve capacity and eNOS-mediated protection may represent one of the key mechanisms underpinning the cardioprotective effects of NO warrants further detailed investigation with larger cohorts of mice and/or mice lacking both eNOS and Nrf2.

## Figures and Tables

**Table 1:** Concentrations (nM) of low-molecular-weight thiols in blood and tissues of wild type (WT) and Nrf2 KO mice (mean ± SD, *n* = 5 per group).

	WT mice	Nrf2 KO mice	*p* value (versus WT mice)
*Heart*			
Total GSH (nM)	144.3 ± 53.11	91.52 ± 12.06	=0.0620 (↓ ns)
GSH (nM)	143.7 ± 52.77	90.77 ± 11.99	=0.0602 (↓ ns)
GSSG (nM)	0.6263 ± 0.427	0.7531 ± 0.352	0.6225
GSH/GSSG	335.0 ± 217.9	140.2 ± 52.51	=0.0887 (↓ ns)
Cys (nM)	16.38 ± 6.976	8.99 ± 1.804	=0.051 (↓ ns)
HCys (nM)	0.6387 ± 0.1949	0.4231 ± 0.06714	↓^∗^0.0475
GluCys (nM)	0.2066 ± 0.03208	0.1852 ± 0.02159	0.2506
Sulfide (nM)	45.9 ± 22.1	28.56 ± 6.597	0.1301
*Aorta*			
Total GSH (nM)	217.2 ± 73.95	105.9 ± 21.91	↓^∗^0.0121
GSH (nM)	216.4 ± 73.54	105.2 ± 22.2	↓^∗^0.0119
GSSG (nM)	1.099 ± 0.2598	0.7146 ± 0.4136	0.1517
GSH/GSSG	224.8 ± 58.4	186.9 ± 101.2	0.5308
Cys (nM)	54.18 ± 12.78	18.15 ± 3.143	↓^∗∗∗^0.0003
HCys (nM)	2.324 ± 0.3268	3.226 ± 2.024	0.3539
GluCys (nM)	0.9574 ± 0.1772	0.6319 ± 0.1166	↓^∗∗^0.0089
Sulfide (nM)	21.51 ± 5.773	16.78 ± 5.570	0.2237
*Liver*			
Total GSH (nM)	8097 ± 833.6	4742 ± 2042	↓^∗∗^0.0094
GSH (nM)	8028 ± 814.8	4592 ± 2025	↓^∗∗^0.0078
GSSG (nM)	68.61 ± 27.77	150.6 ± 43.52	↑^∗∗^0.0075
GSH/GSSG	127.2 ± 31.83	31.55 ± 15.14	↓^∗∗∗^0.0003
Cys (nM)	204.8 ± 16.85	179.1 ± 52.44	0.3272
HCys (nM)	39.12 ± 7.435	18.18 ± 6.563	↓^∗∗^0.0015
GluCys (nM)	0.7695 ± 0.04889	0.5463 ± 0.1343	↓^∗∗^0.0082
CysGly (nM)	96.75 ± 16.4	62.13 ± 21.8	↓^∗^0.0219
Sulfide (nM)	13.19 ± 2.683	15.17 ± 4.521	0.4229
*Plasma*			
Total GSH (nM)	27,144 ± 8464	16,594 ± 3540	↓^∗^0.0331
GSH (nM)	26,456 ± 8364	16,194 ± 3482	↓^∗^0.0351
GSSG (nM)	688.3 ± 171	399.9 ± 93.14	↓^∗^0.0107
GSH/GSSG	39.04 ± 10.17	41.28 ± 7.372	0.7005
Cys (nM)	11,900 ± 2432	8207 ± 1249	↓^∗^0.0165
HCys (nM)	337 ± 159	209.2 ± 35.86	0.1177
GluCys (nM)	668.8 ± 140.9	320.1 ± 83.34	↓^∗∗^0.0014
CysGly (nM)	725 ± 186	1422 ± 728.2	= 0.0751 (↓ ns)
Sulfide	332.1 ± 46.77	457.7 ± 386.6	0.4912
*Erythrocytes*			
Total GSH (nM)	3.94^∗^10^6^ ± 0.52^∗^10^6^	3.77^∗^10^6^ ± 0.30^∗^10^6^	0.5570
GSH (nM)	3.93^∗^10^6^ ± 0.52^∗^10^6^	3.77^∗^10^6^ ± 0.30^∗^10^6^	0.5583
GSSG (nM)	1058 ± 103.7	781.5 ± 153.3	↓^∗^0.0103
GSH/GSSG	3766 ± 726.2	4946 ± 841	↑^∗^0.0449
Cys (nM)	26,254 ± 10,655	18,796 ± 2980	0.1702
HCys (nM)	754.3 ± 317.9	550.8 ± 104.8	0.2109
GluCys (nM)	286.7 ± 118.3	202.7 ± 44.78	0.1757
CysGly (nM)	11,488 ± 5053	11,772 ± 3183	0.9178
Sulfide (nM)	387.3 ± 69.59	490.7 ± 98.66	0.0917

ns - not significant; ↓ - decreased; ↑ - increased; ^∗^ *p* < 0.05, ^∗∗^ *p* < 0.01, ^∗∗∗^ *p* < 0.001.

**Table 2:** Concentrations of nitrite, nitrate, nitrosation products (RXNO), and nitrosyl heme (NO heme) in blood and different tissues of Nrf2 KO mice as compared to WT mice (mean ± SD, *n* = 5 each).

Metabolite	Concentration	WT mice	Nrf2 KO mice	*p* value (versus WT mice)
*Heart*				
Nitrite	*μ*M	1.673 ± 0.4005	0.7784 ± 0.8500	**↓** ^∗∗^0.0039
Nitrate	*μ*M	57.30 ± 40.04	31.22 ± 29.14	ns
RXNO	nM	144.1 ± 26.76	177.4 ± 57.65	ns 0.0603
NO heme	nM	103.8 ± 42.02	79.40 ± 4.777	ns
*Aorta*				
Nitrite	*μ*M	146.1 ± 99.19	87.49 ± 15.87	ns
Nitrate	*μ*M	2755 ± 1166	8428 ± 4852	ns
RXNO	nM	2506 ± 836.9	2712 ± 1154	ns
NO heme	nM	nd	nd	—
*Liver*				
Nitrite	*μ*M	0.2970 ± 0.1423	0.3772 ± 0.3052	ns
Nitrate	*μ*M	28.63 ± 9.395	37.17 ± 9.736	ns
RXNO	nM	1040 ± 136	710.1 ± 278.8	↓^∗∗^0.0019
NO heme	nM	300.0 ± 67.34	162.6 ± 30.52	↓^∗∗^0.0032
*Lung*				
Nitrite	*μ*M	0.7026 ± 0.05554	0.9157 ± 0.2999	**↑** ^∗^0.0133
Nitrate	*μ*M	32.40 ± 3.535	38.05 ± 8.71	ns
RXNO	nM	56.56 ± 21.18	42.79 ± 24.02	ns
NO heme	nM	301.6 ± 92.45	275.0 ± 173.8	ns
*Plasma*				
Nitrite	*μ*M	0.9387 ± 0.1351	0.6771 ± 0.1681	↓^∗∗^0.0080
Nitrate	*μ*M	27.33 ± 6.395	26.42 ± 7.61	ns
RXNO	nM	21.25 ± 2.7	8.852 ± 1.215	↓^∗∗∗∗^<0.0001
NO heme	nM	nd	nd	—
*Erythrocytes*				
Nitrite	*μ*M	1.147 ± 0.1761	0.9267 ± 0.1291	ns 0.0545
Nitrate	*μ*M	46.87 ± 12.34	33.75 ± 13.18	ns 0.0514
RXNO	nM	177.8 ± 46.18	213.1 ± 63	ns
NO heme	nM	48.67 ± 15.46	169.0 ± 54.82	**↑**<0.0001

ns - not significant; ↓ - decreased; ↑ - increased; ^∗^ *p* < 0.05; ^∗∗^ *p* < 0.01; ^∗∗∗^ *p* < 0.001.

**Table 3:** Preserved systolic function in Nrf2 KO mice as measured by cMRT (mean ± SD, *n* = 5 each).

Parameter	Units	WT mice (mean ± SD)	Nrf2 KO mice (mean ± SD)	*p* value
BM	(g)	35.39 ± 2.55	33.41 ± 2.32	0.228
Age	(d)	219.50 ± 26.48	217.50 ± 28.49	0.911
HF	(min^−1^)	547.42 ± 49.56	519.17 ± 26.36	0.287
EDV	(*μ*l)	52.03 ± 7.61	51.41 ± 5.80	0.888
ESV	(*μ*l)	15.33 ± 3.98	15.89 ± 2.42	0.792
SV	(*μ*l)	36.70 ± 4.38	35.52 ± 4.11	0.668
EF	(%)	70.86 ± 3.99	69.10 ± 2.81	0.439
HZV	(ml/min)	19.98 ± 2.17	18.47 ± 2.49	0.330
m (D)	(mg)	112.17 ± 16.00	119.62 ± 8.54	0.380
rel. m	(mg/g)	3.19 ± 0.56	3.59 ± 0.23	0.177
dmitt (D)	(mm)	1.09 ± 0.06	1.07 ± 0.06	0.646
dmitt (S)	(mm)	1.59 ± 0.10	1.60 ± 0.07	0.844
WVmitt	(%)	145.35 ± 7.31	149.24 ± 9.58	0.487

## Supplementary Materials

Supplementary Materials Supplementary Table 1: echocardiographic parameters assessed in vivo by high-resolution ultrasound. Data are reported as mean ± SD; *n* = number of mice. Differences between the two groups were calculated by unpaired *t*-test after testing for normal distribution and equal variances; *p* < 0.05 was considered statistically significant and marked in bold.
