# Supplementary material for: Nrf2 Deficiency Unmasks the Significance of Nitric Oxide Synthase Activity for Cardioprotection
Source: Oxid Med Cell Longev. 2018 Apr 30;2018:8309698. doi: 10.1155/2018/8309698 (PMC5952436; doi:10.1155/2018/8309698)
Supplement: Supplementary Materials — Supplementary Table 1: echocardiographic parameters assessed in vivo by high-resolution ultrasound. Data are reported as mean ± SD; n = number of mice. Differences between the two groups were calculated by unpaired t-test after testing for normal distribution and equal variances; p < 0.05 was considered statistically significant and marked in bold. [file 8309698.f1.pdf]

	WT					Nrf2 KO					eNOS KO				
	WT baseline (BL)	WT post I/R	WT+ETU post IR	P- Value post I/R (vs WT BL)	P- Value + ETU post I/R (vs WT post I/R)	Nrf2 KO BL	Nrf2 KO post I/R	Nrf2 KO + ETU post I/R	P-Value BL vs WT BL	P-Value post I/R vs Nrf 2KO BL	P-Value +ETU vs Nrf2 KO post I/R	eNOS KO BL	eNOS KO post I/R	P-Value BL vs WT BL	P-Value post I/R vs eNOS KO BL
**n**	**9**	**9**	**7**			**8**	**8**	**5**				**5**	**4**		
**EF, %**	55.73 ± 1.75	38.14 ± 2.8	36.27 ± 1.8	**< 0.0001**	n.s.	52.98 ± 1.9	52.4 ± 0.6	39.38 ± 2.2	n.s.	n.s.	******< 0.0001**	63.00 ± 3.259	50.34 ± 0.8	**n.s.**	***0.012**
**HR, bpm**	431.5 ± 13.5	457.2 ± 10.9	441.5 ± 18.4	n.s.	n.s.	409.6 ± 11.9	420 ± 15.1	442.7 ± 18	n.s.	n.s.	n.s.	326.4 ± 13.01	358 ± 21.6	*****0.0003**	n.s
**SV µl**	34.50 ± 1.3	33.42 ± 3.7	30.7 ± 2.3	n.s.	n.s.	38.93 ± 1.9	31.9. ± 2.7	35.27 ± 3.6	**n.s.**	**0.0475**	n.s.	42.00 ± 2.969	36.4. ± 2.7	***0.0221**	n.s
**CO, µl/min**	13.09 ± 0.9	14 ± 1.2	13.48 ± 0.9	n.s.	n.s.	15.1 ± 0.7	13.3 ± 1	11.73 ± 1.2	n.s.	n.s.	n.s.	13.67 ± 0.8640	12.9 ± 0.6	n.s.	n.s
**FS, %**	10.2 ± 1.1	10.1 ± 1.7	9.08 ± 1.2	n.s.	n.s.	11.04 ± 1.4	9.94 ± 1.6	7.635 ± 0.9	n.s.	n.s.	n.s.	12.80 ± 1.658	10.1 ± 1.7	n.s.	n.s
**ESV, µl**	35.31 ± 2.7	31.76 ± 3.3	36.02 ± 6.9	n.s.	n.s.	50.06 ± 3.6	47.07 ± 10.3	56.26 ± 22.7	****0.0057**	n.s.	n.s.	27.23 ± 3.218	26.5 ± 2.1	n.s.	n.s
**EDV, µl**	63.56 ± 10.1	67.2 ± 9.3	70.77 ± 10.5	n.s.	n.s.	88.88 ± 10.1	78.96 ± 14.3	89.86 ± 27.7	*****0.0001**	n.s.	n.s.	69.23 ± 4.36	62.9 ± 3.8	n.s.	n.s
**LVPW;s (mm)**	0.8831 ± 0.06					1.047 ± 0.04						1.331 ± 0.07			
**Mitral Flow**															
**E/A**	1.6 ± 0.3	2.01 ± 0.6	2.46 ± 0.53	n.s.	n.s.	2.13 ± 0.15	2.43 ± 0.5	2.4 ± 0.8	****0,0023**	n.s.	n.s.	1,754 ± 0,09	2.43 ± 0.5	n.s.	n.s
**DT, ms**	12.82 ± 0.6	17.36 ± 1.2	17.98 ± 2.7	**0.0087**	n.s.	24.38 ± 6.5	26.46 ± 7.1	32.06 ± 3.7	*****0,001**	n.s.	n.s.	21,99 ± 1,12	26.46 ± 7.1	*****0,001**	n.s
**MPI**	0.41 ± 0.1	0.55 ± 0.2	0.49 ± 0.1	n.s.	n.s.	0.8 ± 0.2	0.78 ± 0.2	0.81 ± 0.2	*****0,0005**	n.s.	n.s.	0,5707 ± 0,025	0.56 ± 0.04	n.s.	n.s
**IVRT, ms**	17.06 ± 1.7	19.45 ± 2	18.24 ± 3.8	**0.0294**	n.s.	21.92 ± 2.7	21.3 ± 3.3	26.75 ± 4.4	*****0,0011**	n.s.	***0,0432**	16,58 ± 0,58	15.5 ±1.5	n.s.	n.s
**IVCT, ms**	10.23 ± 4	10.17 ± 3.6	10.83 ± 3.4	n.s.	n.s.	7.81 ± 2.2	8 ± 2.3	12.7 ± 2.9	n.s.	n.s.	***0,0225**	8,044 ± 0,69	9.5 ± 0.2	n.s.	n.s
